# Perspectives on trauma-informed maternity care for those with a history of child sexual abuse

**DOI:** 10.3389/fgwh.2025.1597924

**Published:** 2025-06-19

**Authors:** Elsa Montgomery, Lucy Duckworth

**Affiliations:** ^1^ Methodologies Division, Faculty of Nursing, Midwifery & Palliative Care, King's College London, London, United Kingdom; ^2^ The Survivors Trust, Rugby, United Kingdom

**Keywords:** trauma-informed care, child sexual abuse, maternity care, e-resource, co-production

## Abstract

Failure to listen has been a recurrent issue for recent users of maternity services in the UK. The need to listen to women has been recognised in successive reports. Listening is particularly difficult when the population is unheard such as those who have experienced child sexual abuse. Despite its prevalence and lasting impact on physical and mental health, care of women who have experienced child sexual abuse is not usually part of healthcare professional or student education. This paper discusses the benefits of trauma-informed care to meet the needs of survivors of child sexual abuse. It also discusses the co-production of an e-resource on trauma-informed care for women and birthing people who have experienced child sexual abuse. The resource addresses the related educational gap for healthcare professionals and enables the powerful words of this silent, hidden population to be heard.

## Introduction

This paper explores perspectives on emotionally-centred maternity care for women and birthing people^1^ who have experienced child sexual abuse. It considers challenges faced both by survivors^2^ receiving care – even when that care is compassionate and responsive – and healthcare professionals providing care to this population. It discusses the need for trauma-informed care and the co-production of an e-resource designed to address the challenges.

In recent years, failure to listen has been a recurrent issue when shortcomings of the UK's maternity services have been investigated. It was a key theme in the report by the All-Party Parliamentary Group on Birth Trauma (1) and prominent in two of the recent high-profile investigations when failings had been uncovered (2, 3). It impacts the maternity service, when lessons from critical incidents are not learned, and service users whose experience can be distressingly poor and result in psychological trauma. An essential action from Ockenden (2) was that maternity services must ensure that the voices of women and their families are heard, something that was reinforced in the response by Renfrew et al. (4). This is clearly an essential aspect of emotionally-centred care, but women and birthing people who have experienced child sexual abuse are a silent, hidden population (5) and therefore are often unheard.

The mental health and health impacts of violence against women and girls is a priority area in the Women's Health Strategy for England (6). Tackling taboos and stigmas and ensuring women are heard were part of the plan. There is recognition in this strategy that new resources may be needed, and it commits to improving accessibility of evidence-based resources for healthcare professionals. This is a welcome commitment as care of those who have experienced sexual abuse has generally not been part of healthcare professional education. How to respond when a history of child sexual abuse is suspected is a cause for concern for many healthcare professionals (7).

As authors of this paper, we come with very different perspectives. LD is a qualified trainer, policy adviser and researcher working for The Survivors Trust. She regularly contributes to legislation changes and research articles. She wrote the “Check with me First” training programme which discusses the importance of trauma informed care within the NHS when working with survivors of sexual violence. LD is also a survivor of childhood sexual abuse and has two young children. The collision of her professional knowledge combined with the re-traumatisation she experienced during pregnancy has meant she is passionate about raising the awareness of trauma informed care for female survivors of sexual abuse during maternity care. EM is a midwifery academic. Her PhD was on the maternity care experiences of women who were sexually abused in childhood and much of her research since has followed on from this. She has learned a lot from the survivors with whom she has worked. She is in awe of the courage of participants in her doctoral work who shared their experiences with her, and the power of their words. Her PhD uncovered multiple layers of silence, and she has felt a responsibility since to ensure the voices of participants in her research are heard.

LD and EM have now been working together for nearly 10 years and have co-produced two resources. One, hosted on The Survivors Trust website, aims to help women and birthing people who have experienced childhood sexual abuse prepare for pregnancy, birth and parenthood (https://thesurvivorstrust.org/research/pregnancy-birth-and-parenthood-after-childhood-sexual-abuse/) (8). The other, discussed in this paper, addresses the gap in maternity care professional education on the subject (7).

## The prevalence and impact of child sexual abuse

As child sexual abuse is hidden from view, it is difficult to get accurate statistics for its prevalence. Data are not routinely collected in England and Wales, but the Crime Survey, recognised as an under-estimate as it only considers abuse experienced before the age of 16, estimated that 11.5% of women are affected (9). A meta-analysis of studies from 16 countries reported a pooled prevalence among women of 24%, with a rate across the studies from Europe of 17% (10). In its latest report, the Centre of Expertise on child sexual abuse estimates that almost 500,000 children are sexually abused every year in England (11).

Although child sexual abuse is unlikely to be current for most survivors in the maternity services, its lasting impact will be. Adverse childhood experiences (ACEs), including child sexual abuse, increase the risk of many common physical and mental health conditions (12). Pregnancy complications and adverse outcomes are more common among women and birthing people who had ACEs (13, 14). Despite these factors, those who have experienced child sexual abuse rarely disclose to healthcare professionals (6, 14) as they fear an inadequate response and judgement on their ability to parent. They are often made to feel the abuse was their fault and describe feelings of shame and being disbelieved if they do attempt to disclose. Some worry they will be referred to social services and lose the care of their children or that their case will be referred to the police against their wishes, which is common practice whenever sexual abuse is disclosed, regardless of current risk.

Even if a survivor has disclosed and is receiving sensitive care as a result, many aspects of care can be reminiscent of abuse and may take both the person receiving care and the care giver by surprise. Triggers are manifold and are not necessarily related to the intimate examinations that many find difficult and that are commonly an integral part of maternity care. The following examples are recounted in work by EM (15) and the names are pseudonyms chosen by the research participants.

Sue was admitted to an antenatal ward during pregnancy for hypertension and was in a four bedded bay. She was scared of the dark and kept the curtains pulled round her bed at night and her light on. The footsteps she could hear as staff approached to turn the light off were a trigger for her:

I'm completely scared of the dark and to lie there with someone walking into your little curtain bit and turn the light off was horrible because you can hear the footsteps coming … and you know, footsteps have a real big meaning when you've been treated not well as a child.

Mia was in labour at home and progressing well, but the actions of the midwife she trusted and to whom she had disclosed, changed that:

…and I could feel the feeling to push and then she did an internal examination then it gave me like a bit of a problem in my head and she got a torch out, which is what he used to do. He used to get like a torch out to look cos it used to be like in a den, sort of thing, so, so she got a torch out and it was dark and I started thinking I needed to go to the hospital.

It was not the vaginal examination that Mia found most difficult here, but the torch, which the midwife used to sustain the calm, dimly lit environment she had created with Mia and which, unbeknown to her, replicated the abuser's action. Mia was admitted to hospital and had an epidural which was a relief for her. However, being confined in bed due to an epidural can also be difficult, even when requested by the woman, as Sam's account shows:

If I'm stuck on a bed, an’ I can't get out, that is just like horrible it is, and then people coming in the room all the time and it, it triggers flashbacks.

The review of maternity services at the Shrewsbury and Telford Hospitals NHS Trust (2) reports recollections from women involving loss of power and control, excessive, painful vaginal examinations and not being listened to, all of which could leave someone with lived experience of child sexual abuse feeling unsafe (16).

These experiences highlight the importance of a universal trauma-informed approach which offers choice and empowers service users to say what works for them, rather than expecting healthcare professionals to know what those in their care may find difficult and what they can do to help.

## Trauma-informed care

A trauma-informed approach recognises the widespread nature of trauma and the barriers it presents in accessing services. Both service users and staff are impacted. The rates of sexual violence may be as high, if not higher for staff than the general population (17). Throughout delivery of the “Check with me First” programme (https://thesurvivorstrust.org/training/checkwithmefirst/) many professionals disclosed their own abuse to LD and cited it as a reason for joining the profession and wanting to help others. The intersection between professionals’ own trauma histories and their ability to deliver trauma-informed care is significant and underexplored in healthcare literature.

The aim of trauma-informed care is to promote feelings of psychological safety, choice, and control, which can be achieved when people feel seen, heard and cared for (18); to prevent re-traumatisation, not to treat the trauma. We know that trauma-informed maternity care can have a positive impact on the experience of women and birthing people (19), but that available education and resources are lacking (20). Renfrew (4) has recognised that education is a key component of effective change. Care of women who have experienced abuse (non-recent and current) was included in the Standards of Proficiency for Midwives for the first time they were updated by the Nursing and Midwifery Council in 2019 (21) so it will now be part of the pre-registration midwifery curriculum in the UK. However, there are many maternity care professionals who have not had education on the subject (6, 22). Our co-produced e-resource on trauma-informed maternity care for women and birthing people who have experienced child sexual abuse recognises this gap. It has been approved for inclusion on the NHS learning hub.

## Development of our co-produced e-resource

Care of women and birthing people who have experienced child sexual abuse can be challenging for personal and professional reasons, especially when professionals need to engage empathetically with service-users whose disclosures resonate with their own lived experiences. Our work to inform the development of the resource confirmed the lack of available education on the subject and how unprepared students and staff can feel to care for those who have experienced abuse (7). This can leave healthcare professionals feeling awkward if they suspect a history of abuse and means they may avoid addressing the situation as they do know how they should respond. “What do they want from us?” was an underlying question from practitioners when contemplating care of those who have experienced abuse (7). Having some sort of checklist would have eased their anxiety but would risk disempowering those in their care.

A trauma-informed approach understands the impact of trauma, recognises when someone is feeling uncomfortable, responds to that discomfort and resists re-traumatisation (23). This can be facilitated by ensuring control rests with the service user who is the expert in what they need. Clear guidance was provided by one of our focus group participants:

I think there as well that's a lack of questioning, you know, it's going back to this “oh we've done our training, we know what you as a survivor want” and actually they need to just ask (7).

Renfrew recognises that “a diversity of voices from women themselves in the education and training of health professionals” is needed (4 p2). This feels particularly important when the population is hard to hear. Our resource is clear from the start that users will not get to the end with a set of rules to apply, but they will have heard powerful words from those with lived experience of child sexual abuse and maternity care. However, there were under-represented voices in our work who also need to be heard. All the women who participated in EM's PhD were white. In the work we have done since, Black women and women of colour were under-represented. The latest MBRRACE report (24) continues to present a stark picture of the inequities faced by these populations in the maternity services. Ahead of making the resource widely available across England, we needed to be sure that it reflects the experiences of these under-represented women.

A number of workshops, co-facilitated by members of the relevant communities were therefore convened, both in person and online. An online workshop was particularly important for Muslim women who did not wish to be identifiable in their communities. In this workshop, women were given the option to leave their cameras off and write in the chat, rather than allowing their spoken voice to be heard. An in-person and an online workshop were convened with Black and People of Colour by “Little Ro”, a survivor-led community dedicated to amplifying the voices of Black survivors. During these sessions, the resource was presented, and the voices of those with lived experiences of abuse were not just heard but valued. Their stories were shared openly, shedding light on the challenges they face. While the participants recognised their experiences reflected in the resource, they expressed that more work is needed. Specifically, there is a crucial need to create a dedicated space where the voices of Black and People of Colour are not only heard but centred, allowing them to speak directly to their unique and often overlooked experiences within the system and how overcoming those experiences can be managed.

## The way forward

Discussions between Little Ro and EM on the production of resources to ensure the perspectives of Black and People of Colour are duly respected have started. In NHS trusts in which LD has provided #CheckWithMeFirst training, 100% of delegates said it should be mandatory for all maternity healthcare professionals. This view reflects the report by The All-Party Parliamentary Group on Birth Trauma (1) which recommends mandatory training on trauma-informed care, and that awareness of the causes and impact of birth trauma should be a mandatory part of midwifery and obstetrics education and training. Work to test the implementation of the resource discussed in this paper in the NHS and midwifery pre-registration programmes in England is in preparation.

## Discussion

Recurrent reports have highlighted failure to listen as a problem in the maternity services, but the importance of ensuring women's voices are heard is now part of policy. There are particular challenges when working with populations who are hard to hear, such as those who have experienced child sexual abuse. We have suggested that trauma-informed care is an appropriate way to respond to these challenges and have discussed the co-production of an e-resource that addresses the need for maternity care professional education on the subject. Trauma-informed care is a process which is heavily influenced by the professionals’ own life experiences and stressors. It recognises that every person, whether professional or service-user has a unique experience and therefore requires a unique response which cannot not be directed in any learning or text. There is no linear response from trauma, and as such there should be no linear treatment of it. We advocate for all professionals being given the tools to offer bespoke care, regardless of whether a disclosure of sexual violence has been made or not, and not to be restricted by guidance which does not recognise previous experiences of the professional or patient.

## Data Availability

The original contributions presented in the study are included in the article, further inquiries can be directed to the corresponding author.

## Footnotes

^1^We recognise that not all birthing people identify as women. However, when we refer to participants in our studies who do, our language will reflect theirs out of respect for them.

^2^We recognise that not all women who have experienced child sexual abuse find the term ‘survivor’ relevant or helpful, but we use it as this was the preference of most of the participants in our work.
